# Design and Construction of a Bilateral Haptic System for the Remote Assessment of the Stiffness and Range of Motion of the Hand

**DOI:** 10.3390/s16101633

**Published:** 2016-10-01

**Authors:** Fabio Oscari, Roberto Oboe, Omar Andres Daud Albasini, Stefano Masiero, Giulio Rosati

**Affiliations:** 1 Department of Management and Engineering, University of Padova, Stradella S. Nicola 3, 36100 Vicenza, Italy; fabio.oscari@unipd.it (F.O.); roberto.oboe@unipd.it (R.O.); 2 Center for the Development of Nanoscience and Nanotechnology, Universidad de Santiago de Chile, Av. Lib. Bernardo O’higgins, 3363 Santiago, Chile; omar.daud@usach.cl; 3 Department of Neuroscience, Universiy-General Hospital of Padova, Via Giustiniani 2, 35128 Padova, Italy; stef.masiero@unipd.it

**Keywords:** rehabilitation robotics, force feedback, remote rehabilitation, series elastic actuator, tele-assessment

## Abstract

The use of haptic devices in the rehabilitation of impaired limbs has become rather popular, given the proven effectiveness in promoting recovery. In a standard framework, such devices are used in rehabilitation centers, where patients interact with virtual tasks, presented on a screen. To track their sessions, kinematic/dynamic parameters or performance scores are recorded. However, as Internet access is now available at almost every home and in order to reduce the hospitalization time of the patient, the idea of doing rehabilitation at home is gaining wide consent. Medical care programs can be synchronized with the home rehabilitation device; patient data can be sent to the central server that could redirect to the therapist laptop (tele-healthcare). The controversial issue is that the recorded data do not actually represent the clinical conditions of the patients according to the medical assessment scales, forcing them to frequently undergo clinical tests at the hospital. To respond to this demand, we propose the use of a bilateral master/slave haptic system that could allow the clinician, who interacts with the master, to assess remotely and in real time the clinical conditions of the patient that uses the home rehabilitation device as the slave. In this paper, we describe a proof of concept to highlight the main issues of such an application, limited to one degree of freedom, and to the measure of the stiffness and range of motion of the hand.

## 1. Introduction

Stroke is the third leading cause of death after cardiovascular diseases and cancer and represents the greatest cause of disability and impairment in the industrialized world [1]. The damage to the central nervous system caused by stroke can lead to impaired motor control on the affected side (hemiparesis). The available scientific literature suggests that the earlier is the rehabilitative intervention (as well as an intensive and prolonged multisensory stimulation), the more effective is the functional recovery [2,3].

The conventional approach to upper limb impairment can be efficaciously integrated by using properly-designed robots that have a proven high effectiveness in promoting recovery [4,5]. Particularly, haptic technology constitutes a powerful tool for developing active training devices. Patients under treatment can be stimulated in several ways, ranging from passive mobilization to sophisticated interactions with the virtual world. These can be delivered by different types of feedbacks (haptic, visual, auditory, tactile, cutaneous), through computer screens, headphones and other systems [4,5,6,7,8,9,10,11]. In a standard scenario, a patient interacts with multi-feedback haptic devices in a rehabilitation center, following a precise medical therapy program. The devices used by the patients act as modern, effective and safe tools to reproduce motor and functional learning experience. These can perform intensive intervention; they can monitor the improvement recording kinematic/dynamic data (e.g., joint angles, forces, etc.), giving some kind of score related to patient performance, adjusting the intervention to patients’ progress [5,6].

However, as Internet access is now available at almost every home, the idea of moving robotic rehabilitation from the hospital to the house is gaining a wide consent. On the one hand, it bring a medical care program to the patient’s house where it could be carried out more comfortably. On the other hand, this type of rehabilitation can reduce the hospitalization time of the patient and, thus, therapy costs [12].

The systems that are able to provide rehabilitation at home are called tele-healthcare or tele-medicine systems [13]. They consist of: a home rehabilitation device (similar to those of the hospital), a central server at the hospital and a system management controller (split between home device and remote central server). Rehabilitation programs are synchronized with the home rehabilitation device by means of the system management controller that allows the home device to download information from the central server. The home device deals with setups that allow patients to train several hours a day, by performing the pre-programmed exercises at home, involving force feedback as a response, while interacting with a virtual environment. Finally, the system management controller monitors safety, helps the patient to solve possible issues and records the measurements of kinematic/dynamic parameters and performance scores during patient training. These data are then sent to the central server that could redirect information (even in real time) to the therapist laptop or smartphone, in order to monitor patient progress [13,14]. Since the therapist cannot interact with the patient, this type of applications is also called unilateral tele-rehabilitation (tele-rehabilitation can be defined as teleoperation in rehabilitation).

The controversial issue of tele-healthcare is that the recorded data of the therapy sessions of the patients do not actually represent the clinical conditions of their limbs. The assessment of the clinical conditions, in fact, involves well-defined clinical tests that have to be performed by clinicians, according to the medical assessment scales, as, e.g., the Fugl–Meyer Assessment scale (FMA) [15] and the Medical Research Council scale (MRC) [16]. Since the outcomes of such tests are the only ones providing to clinicians a direct and immediate feeling on patient conditions and, also, the degree of effectiveness of the exercises proposed, a patient should frequently go to the hospital to undergo them. Moreover, the therapists could need a tight, physical interaction with the patient with the impaired limb in the rehabilitation device to also finely trim the exercise, in order to maximize its effectiveness, mostly for the treatment of patients in the acute or sub-acute phase. Therefore, tele-healthcare is not able to perform a fully-remote rehabilitation program.

To answer the issue described above, especially the one related to a tight interaction, we propose the use of a bilateral master/slave haptic system. Such a system can allow the clinician, who interacts with a master haptic device, to assess remotely and in real time the clinical conditions of the patient that uses the home rehabilitation device, extended to the additional use as a slave device. This paper presents a proof of concept of such a system, with single-d.o.f. (degree of freedom) devices, to measure the stiffness and the range of motion of the hand, which are two parameters considered in the traditional clinical tests. The system was designed, constructed and preliminarily tested on healthy subjects. This study could represent a first analysis to highlight the main issues that must be dealt with, in order to create a system for the potential application case of the remote assessment of patients with impaired fingers of the hand. In the proposed system, control schemes have been made use of that stabilize the bilateral interaction, even in the case of variable network performance (as those for the Internet), in order to achieve safe operations.

To date, there are only some contributions presented in the literature that are focused on the system design of bilateral interaction for potential applications in real-time clinical remote assessments of impaired limb (and without the design of an ad hoc slave device). Preliminarily, some indications were provided by Park et al. [17,18] who described an example of the design of a tele-assessment system for the evaluation of elbow spasticity on patients with neurological impairments and showed some preliminary results. However, they focused on the design of an ad hoc portable system, and they used a simple control strategy that did not allow the system to deal with variable network performance, so the remote assessment of the degree of spasticity was performed by using a “record and replay” strategy, i.e., the spasticity test was automatically performed at the patient’s side and later replayed at the therapist side. More recently, some works have been addressing the problem of real-time bilateral tele-rehabilitation that considers at least the real-time scenario in which the therapist could guide a rehabilitation robot that imposes the motion of a second robot that is remotely used by the patient. Lanini et al. [19] presented a teleoperation system for two six-d.o.f. ARMin arm skeletons for tele-rehabilitation, which makes use of compliance control and torque feedback. Zhang et al. [20] proposed and preliminarily tested a system focused on elbow joint motor recovery. The system consists of a bilateral control scheme, a human-upper-limb-like device as the master device for the therapist and an exoskeleton device as the slave device. Both devices are characterized by the use of elastic elements, guaranteeing a compliance control of the telerehabilitation system.

Other reports on these concepts are related to more general bilateral master-slave tele-rehabilitation robotic systems, as the one proposed by Chiri et al. [21,22] that consisted of a glove acting as the master for the therapist and a powered hand exoskeleton acting as the slave rehabilitation device for the patient. A similar approach has been reported in Farulla et al. [23], but using a vision-based pose estimation, which makes the system more intuitive for untrained personnel. However, in both cases, the focus was remote rehabilitation with a bilateral interaction to allow the therapist to adjust the task of the patient based on a real-time feedback. In fact, clinical hand tests, as according to the medical assessment scales, could not be performed with such equipment.

The paper is organized as follows. In Section 2, all of the components of the tele-operation system are described, as well as the required specifications. In Section 3, the control-system design is explained, particularly the position-force control architecture. A study of such a system, in terms of transparency, stability and performance, is then presented in Section 4. Analytical results are then compared with some preliminary experimental results in Section 5; particularly, the evaluation of the maximum perceived stiffness is a key issue for the actual tele-robotic application. Finally, conclusions are drawn in Section 6.

## 2. Specifications and System Description

**Figure 1:**
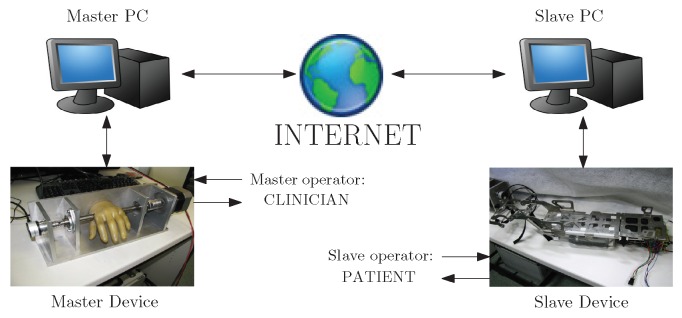
A simplified scheme of the teleoperation control system architecture.

The main goal of the proposed tele-assessment system is to implement a bilateral interaction between two haptic devices (master and slave). These devices are connected through a data network (e.g., Internet), as shown in Figure 1. In the possible application of such a system for the clinical assessment of the hand, the master operator would be the clinician; vice versa, the patient would represent the slave operator.

A task-oriented design approach for both master and slave devices usually yields dedicated system solutions. A more flexible solution, which is highly desirable, could be achieved by combining a master device dedicated to the clinical assessment, with a slave device suitable not only for the remote assessment, but also for stand-alone rehabilitation. Actually, this solution could extend the possibilities of the post-stroke therapy program, usually made of periodical assessments followed by treatments, achieving a fully-remote rehabilitation program [24].

Taking into account these considerations, a one-d.o.f. prosthetic hand (master device) was interfaced with an existing active one-d.o.f. orthosis (slave device), designed for a stand-alone hand rehabilitation of post-stroke patients.

Since our aim consists of highlighting the main issues concerning a bilateral tele-assessment system and we focus here on the measurements of the state of the hand, we can say that considering devices with one d.o.f. is not too limiting for this type of study. In fact, the results of several authors have shown that simple devices, such as single-d.o.f. hand devices, can be enough for a basic rehabilitation of the fingers in severe to moderate stroke patients. For example, the hand module of the Gentle-G [25] includes one actuator for the thumb and two for the four fingers together. The Howard [26] has one actuator for the thumb, one for the four fingers together and one for the wrist. The IntelliArm [27] (hand module) was designed to drive the hand to open/grasp at the metacarpophalangeal (MCP) and thumb joints with one d.o.f only. The tele-assessment system that is presented here is based on the same concept: a single-d.o.f. mechanism is used to actuate the flexion/extension of the four fingers together about the MCP joint.

### 2.1. Specifications

A person who has suffered neurological disorders commonly has a reduced range of motion (ROM), as well as muscular weakness and spasms. The clinician must evaluate these situations, in order to define the therapy program. Performing a remote assessment of impaired hands with a haptic teleoperation system should always hold the same clinical criteria of a conventional assessment. With this aim and in collaboration with the Unit of Rehabilitation at the Hospital of Padua, we have examined the main clinical tests for patient’s hand. According to the most frequently-used stroke assessment scales, (the Fugl–Meyer Assessment scale (FMA) [15] and the Medical Research Council scale (MRC) [16]), five tests can be considered as suitable for the remote clinical evaluation of the fingers on the hand:

- Passive range of motion (ROM) test: at the beginning of the evaluating session, the clinician slowly moves the fingers on the patient’s hand (to minimize the potential-reflected response) to find the range of motion.
- Active ROM test: the patient is asked to move the fingers on the hand up to their moving limit.
- Muscular resistance test: the patient is asked to keep the fingers on the hand fixed, while the clinician tries to either flex or extend them.
- Muscular force test: the patient is asked to either flex or extend the fingers on the hand, while the clinician tries to keep them blocked.
- Spasticity test with catch angle evaluation: the clinician holds the patient’s hand and moves their fingers at different velocities in order to feel the velocity-dependence of the resistance torque and the ‘catch’, defined as the angle at which the resistance to a movement abruptly grows.

Even if we limited the study to analyzing issues concerning tests of the range of motion and muscular resistance/force only, to perform all of them, the bilateral tele-assessment system should comply with the following technical specifications:

- Patient’s hand position sent back to the master (ROM test);
- Fair reproduction of force (muscular/resistance test);
- Maximum transparency at each side;
- Stability in the presence of, at least, small network delays (standard ADSLin a limited range);
- Maximum versatility of the slave device, in order to guarantee an effective two-fold use of the active hand orthosis (remote assessment and rehabilitation).

### 2.2. Master Device

The master device was designed in order to provide the most realistic sensation, either physical or psychological, to the therapist, who should be remotely evaluating the condition of the patient’s hand.

**Figure 2:**
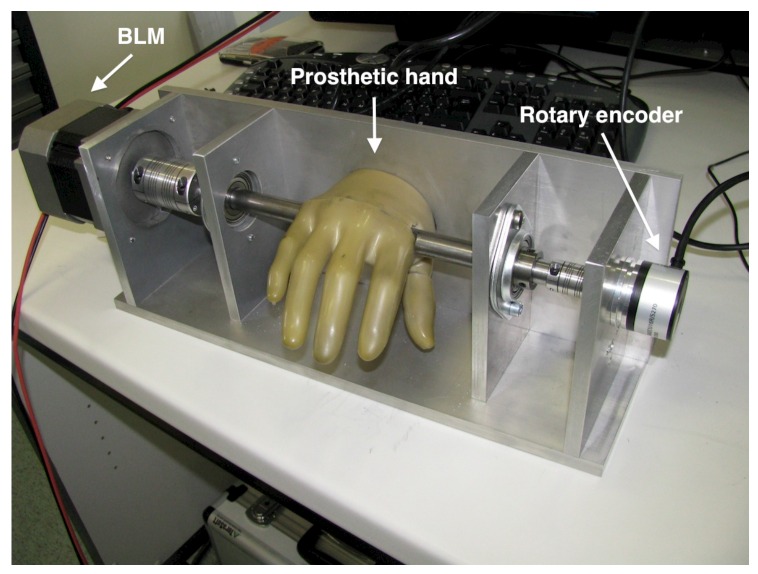
The prosthetic-hand master device.

The end mechanism is formed by a prosthetic hand, drilled at the level of the joints, to which metacarpal bones and phalanges are connected. The palm and the thumb are fixed to the reference frame, while the four fingers rotate together with the shaft. The shaft itself, supported by two bearings, is actuated by a brushless motor MB 082 GA210 with 2.8-Nm peak torque, which is driven by a PWM current amplifier (Microstar SMB60 10/20 ARM: 20-A peak current), and an ELTRA 20,000-pprresolution incremental encoder measures its angular position. The maximum force at the fingertip is around 30 N, which is in the range of the maximum force applied by the orthosis (the slave device) on the patient’s hand. Figure 2 shows the mechanism, where the fingers can move in a range of about $40^{\circ }$. The master device is controlled via PC and MATLAB/Simulink, using a data acquisition board (PCI Multifunction I/O Sensoray 626). All variables are sampled at 1 kHz.

### 2.3. Slave Device with VS-SEA

**Figure 3:**
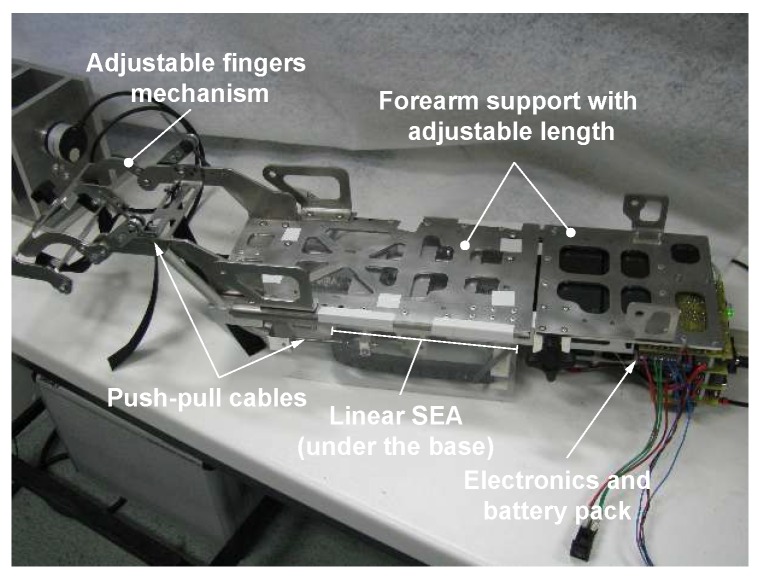
The slave orthosis.

An actuated orthosis, shown in Figure 3, was used as a single-d.o.f. slave unit of the teleoperation system. The device was designed for the rehabilitation of the fingers on the hand in patients with ischemic and/or hemorrhagic stroke outcomes [28].

The chassis of the slave hand orthosis consists of a stainless steel plate, on which the patient’s forearm is tied. The plate also carries a linear series elastic actuator (SEA) block, a mechanism for moving the fingers, the control electronics and the power supply.

**Figure 4:**
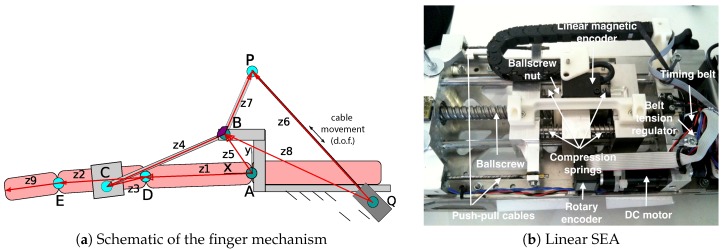
Details of the slave orthosis. (**a**) Schematic of the whole mechanism (gray-black) connected to the finger (pink), which shows the rigid links, the revolute joints and the slider (point *Q*). Joints with a darker color are fixed to the frame. (**b**) Identification of the components included in the linear series elastic actuator (SEA).

Let us consider the motion of the fingers in a grasp/release exercise as a simple planar movement, by neglecting the abduction/adduction. As shown in Figure 4a, the fingers acting together can be considered as a single finger, made of three links (the phalanges: $z_{1}$, $z_{2}$, $z_{9}$) and three route joints (*A*, *D*, *E*). The fingers are moved by means of the four-bar linkage ABCD, representing the main mechanism of the orthosis. The proximal phalanx $z_{1}$ acts as one of the links of such a mechanism, while the intermediate phalanx $z_{2}$ is related to the second link $z_{3}$, which is rigidly rotated with respect to $z_{2}$. A third link $z_{5}$ rigidly connects the metacarpus to the forearm support (frame of the mechanism). The last link $z_{4}$ is connected to the frame and to the intermediate phalanx by means of a phalanx support (gray color). The length of this last vector is variable in order to fit different hand sizes. The mechanism is driven by a tendon $z_{6}$ that, sliding into the pipe in *Q*, pushes or pulls the link $z_{7}$ that is rigidly connected to $z_{4}$. The rigid body represented by $z_{4}$ and $z_{7}$ works as the crank link of the four-bar linkage; the link $z_{8}$ represents instead the frame of the drive mechanism QPB. In the mechanical design of the mechanism, $z_{6}$ was implemented by means of two push-pull flexible cables, enclosed in bent aluminum pipes, as shown in Figure 3.

The cables are moved by means of the linear SEA [29], as shown in Figure 4b. It consists of a solution with an elastic element placed between the motor and the load, whose displacement is measured to implement a good force control with minimum impedance [30]. The use of SEA enhances safety, thanks to a highly compliant behavior (a desirable feature for robotic therapy) [31], in addition to provide a good impact tolerance (necessary to avoid injury to the patient) and a high force/mass ratio [30,32]. In this mechanical solution, a velocity-controlled DC motor (motor Maxon Motor 354344: 29 Nmm of continuous torque; encoder Maxon Motor HEDL 5540: 500 ppr) drives a miniature ball screw with a 12.7-mm lead via a transmission belt (1:3.6 transmission ratio). The nut of the ball screw is fastened to one end of four springs in parallel connection (1290 N/m spring stiffness for each), whose opposite ends push the tendons that drive the mechanism, as shown in detail in Figure 4b. A SIKO linear encoder with 200-ppmm resolution is used to measure the displacement of springs as an indirect measure of tendon force. With this configuration, a maximum fingertip force of 30 N can be reached over a maximum metacarpophalangeal (MCP) joint rotation of nearly $60^{\circ }$.

As detailed in the next section, the SEA is here implemented by a combination of an inner velocity loop, provided by the motor driver, and an external force control loop, implemented in a Microchip dsPIC microcontroller. The overall control strategy results in a velocity-sourced SEA (VS-SEA) [33]. Wyeth [33] showed that the VS-SEA as implemented in the orthosis (outer force loop with inner velocity loop) has well-defined characteristics that the improve safety and performance over conventional high impedance actuators and traditional SEA systems (simple force loop), which show instead a detriment of the system performance.

### 2.4. Communication Line and Safety

An effective bilateral interaction for remote rehabilitation requires a real-time performance of the network, which must provide a high frequency data exchange. In order to set up the communication between the clinician and the patient, a bandwidth estimation and data flow handling between master and slave stations are needed. This problem has been addressed by one of the authors [34]; hence, the same procedure is used to estimate the maximum allowable data rate between master and slave. A lost data packet can be detected by using a simple sequential numbering for each sent packet, while round trip delays are obtained by comparing timestamps with a local clock. All measurements and detection features are supported within an enhanced-UDP protocol, as described in [34].

The proposed application is conceived of only for a limited distance between the slave station (patient site) and the diagnostic center, from where the clinician could control one or more patients. Measurements in short range connections (within 100 km) conducted in Italy show that even a standard ADSL connection achieves a 25-ms round trip delay (RTT), with a 400-kbit/s sustained upload rate [35], which allows data sampling at several hundreds of Hertz. Such a high rate, however, is not actually necessary in the proposed system. In fact, this system is used mostly in assessing the characteristics of the patient’s hand, which is perceivable in a low-frequency range. This issue relaxes the requirements for the underlying network connection, in terms of throughput and jitter.

In the proposed system, data are sent through the IP connection every 10 ms, while the actual observed jitter is around 3 ms [35]. As for the variable delay, it has been handled with a simple buffering procedure. It provides a constant end-to-end delay, with a small drawback due to the slight increase of the communication delay. On the other hand, having a constant delay makes the analysis of the overall stability more treatable.

From the point of view of safety, as reported in Section 2.3, the mechanical structure of the orthosis has been conceived of to limit excessive forces at the patient’s hand. Besides, the patient is tightly harnessed with adjustable belts to the orthosis. Additional safety has been achieved by implementing the force control loop at the patient’s side of the bilateral system. Furthermore, as missing data packet in the communication line can destabilize the force control loop at the patient’s side, the missing data packets are then replaced by null packets. This guarantees that the last command force received is used in the force control loop, so an interruption in the communication link cannot harm the patient. Safety was also preliminarily tested, as shown in Section 5.

## 3. Control-System Design

### 3.1. Definition of the Control Architecture

As explained in Section 2, the proposed bilateral system is composed of a dedicated master device interfaced by an already available stand-alone rehabilitation system, which was modified in order to work as the slave device, as well. This solution led to some constraints in choosing the control strategy. Moreover, several control specifications must satisfy the functional objectives of the system.

At the slave site, the hand orthosis has a well-defined mechanical structure, designed for integrating an SEA architecture that improves the performances by implementing a control scheme. It has also a velocity loop nested into a force control loop (the so-called VS-SEA system) [33]. Therefore, the VS-SEA structure, implemented in the hand orthosis, has proven to be a constrained solution for the slave control block to ensure safety and good performances in both tele-assessment and robotic rehabilitation (see the last specification in Section 2.1). Thus, a suitable tele-operation control scheme, should consider the force reference sent forward from the master device. Then, based on the slave structure, we considered a two-channel control architecture. This architecture is simple, in such a way that it can relax the communication requirements. Furthermore, it requires a fewer number of sensors. It is easy to analyze.

A two-channel control architecture with a force channel from the master to the slave can only assume two configurations (the schemes for a two-channel teleoperation system are considered as denominated by the variable, which is measured and sent to the opposite remote site respectively from slave and master), that is force-force (F-F) or position-force (P-F). However, due to the specifications about the ROM clinical test, the patient’s hand position should be sent back to the master. This has led to a P-F controller. Specifically, since a position controller with the patient’s hand coordinate as the reference at the master side would guarantee safe movements at the patient’s side, we implemented this control mode, known as “admittance mode haptic interface” [36,37].

Considering all of the above considerations, a transparency-optimized control law was implemented in a two-channel P-F tele-operation scheme with admittance mode, also satisfying the maximum-transparency specifications.

**Figure 5:**
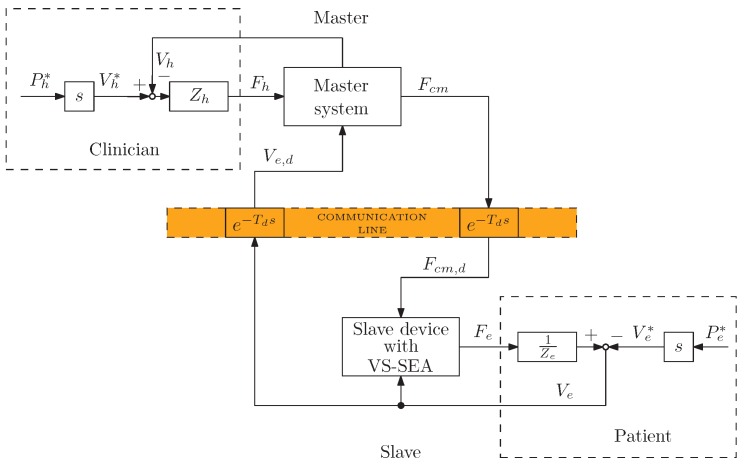
The teleoperation control system architecture.

Figure 5 shows the block diagram of the defined architecture. $T_{d}$ indicates the communication-channel delay, whereas *V*, *F* represent velocity and force, respectively, and *Z*, *Y* impedance and admittance. It is worth noticing that the proposed P-F architecture is slightly different from the standard P-F teleoperation system (see Figure 5), since the master command force $F_{cm}$ is sent to the slave site, instead of the master contact force $F_{h}$. However, this choice does not compromise the analysis, as long as it remains in a bounded range of operation.

### 3.2. Control Design: Four-Channel and Two-Port Network Models

In the literature, several methods and frameworks have been outlined to both design and analyze teleoperation systems. In this study, we employed the two-port network approach to find here a flow-effort representation of the system, similarly as used in the electrical networks [38,39,40], necessary to derive the transparency-optimized teleoperation control law through the master-slave two-port network (MSN) matrix.

**Figure 6:**
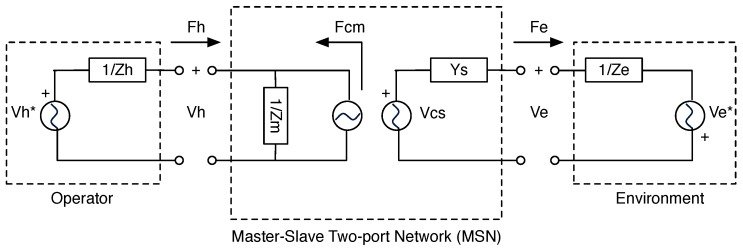
Master-slave two-port network (MSN) block diagram.

Figure 6 shows the two-port representation of the P-F teleoperation system illustrated above. At the input port (master port), the MSN block interacts with the operator (therapist); at the output port (slave port), the MSN interacts with the environment (patient). At each port, the “flow” variables are respectively $F_{h}$, $F_{e}$, and the “effort” variables are $V_{h}$, $V_{e}$.

In Figure 6, the master device was modeled as an impedance system, whereas the slave device as an admittance system, treating the motor as a velocity generator instead of a torque generator, as suggested by Robinson et al. [29] and Zaad et al. [41]. In addition, it is worth noticing that both the clinician’s and patient’s dynamic models were considered. Referring to Figure 6, the models of the clinician (operator) and the patient (environment), respectively, are represented in terms of force and velocity by these equations:

(1) $$\begin{array}{ccc} F_{h} & = & Z_{h}\left(V_{h}^{*}-V_{h}\right)=\left(c_{h}+k_{h}/s\right)\left(V_{h}^{*}-V_{h}\right) \end{array}$$

(2) $$\begin{array}{ccc} V_{e} & = & F_{e}/Z_{e}-V_{e}^{*}=Fe/\left(c_{e}+k_{e}/s\right)-V_{e}^{*} \end{array}$$

where, neglecting the neural feedback, the impedances of the hand $Z_{h}$ and $Z_{e}$ represent only the dynamics of the muscular contraction and the passive tissues, which, in a passive model of a limb, outlined as a “position generator” [42,43], may be defined as a damper-spring system with stiffness *k* and viscosity *c* [37,44].

Considering $F_{h}$ and $-V_{e}$ as the input variables, its dynamic can be represented by the inverse hybrid matrix G:

(3) $$O_{g}=\left[\begin{array}{c} V_{h}\\ F_{e} \end{array}\right]=\left[\begin{array}{cc} g_{11} & g_{12}\\ g_{21} & g_{22} \end{array}\right]\left[\begin{array}{c} F_{h}\\ -V_{e} \end{array}\right]=G\cdot I_{g}$$

**Figure 7:**
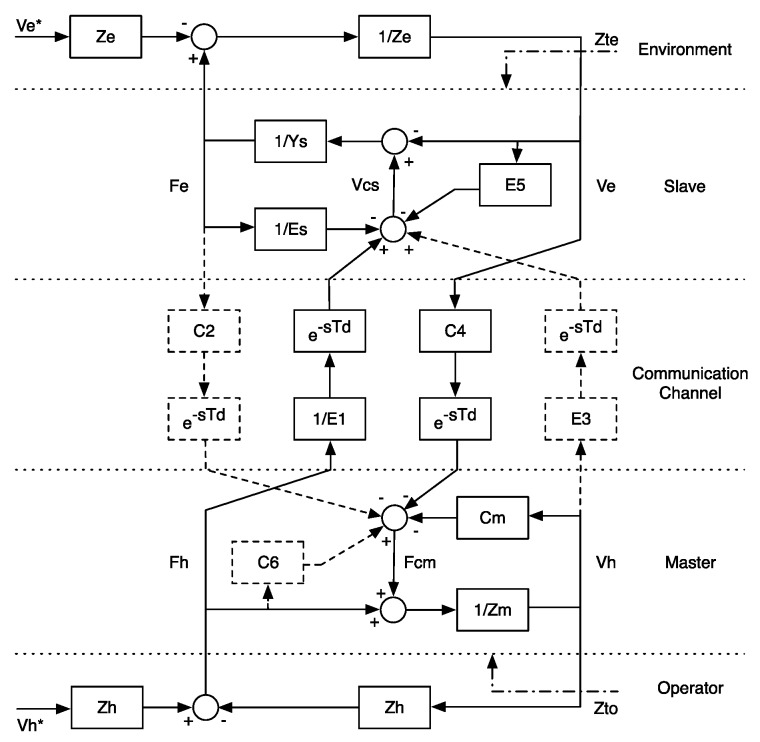
Block diagram of an impedance-admittance four-channel teleoperation system. In dashed lines, the non-used channels in the position-force (P-F) type control architecture.

In order to compute G, it is necessary to obtain both the master and the slave control action ($F_{cm}$ and $V_{cs}$, respectively). For this purpose, we rearranged the tele-operation system of Figure 5 in order to get an impedance-admittance type of a two-channel P-F architecture, by relying on a simplified version of a generic four-channel bilateral controller [41]. As shown in Figure 7, the dashed lines and blocks represent the parts that were not implemented in the control scheme. $T_{d}$ indicates the communication-channel delay, whereas *C*, *E* represent the transfer functions of the control actions and *V*, *F* velocity and force, respectively.

However, the block diagram of Figure 7 differs from the one of Figure 5, as the force coordinate sent from the master to the slave side is $F_{h}$, instead of being $F_{cm}$. This difference is addressed in Equation (6).

**Figure 8:**
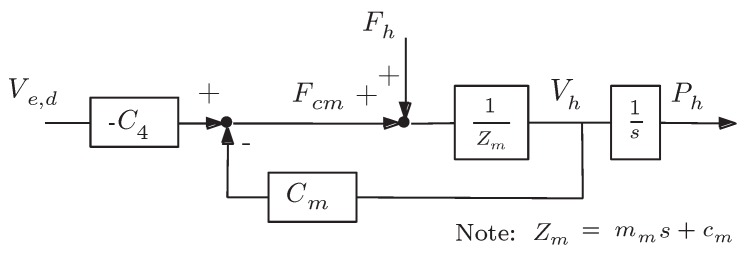
Control block diagram of the master.

**Figure 9:**
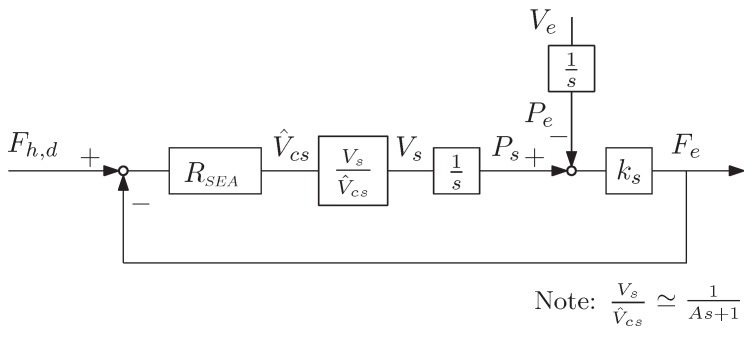
Control block diagram of the slave.

By using the same symbols of Figure 7, the resulting control block diagrams of the master and the slave are shown in Figure 8 and Figure 9, respectively. The master is an impedance device (impedance $Z_{m}$) controlled by a position regulator (Figure 8). It follows the delayed position of the slave $P_{e,d}$ by means of force command $F_{cm}$, which is generated by both the master local position controller $C_{m}$ and the slave coordinating force feedforward controller $-C_{4}$; then, it applies a force $F_{h}$ to the environment with which it is in contact, in response to the measured position $P_{h}$ [45].

At the slave side, a VS-SEA control structure was imposed, shown in Figure 9. Here, the slave local force controller ${E_{s}}^{-1}$ is fixed equal to the master coordinating position feedforward controller ${E_{1}}^{-1}$, achieving the external force loop, which is regulated by the SEA controller $R_{\mathrm{SEA}}$. The external force loop generates the velocity reference ${\hat{V}}_{cs}$ that, in free motion, is only due to the delayed master contact force $F_{h,d}$ (used as the force reference), and in constrained motion, it goes to zero as the measured slave contact force $F_{e}$ converges to $F_{h,d}$. Herein, a force control at the patient’s hand is achieved. Moreover, it was assumed that friction and the equivalent reduced-to-end-effector mass of the slave device were low and/or negligible. Actually, the transition from rate control to force control follows the naturally-transitioning rate to force control (NTRFC) concept [46], as similar to those implemented in [37].

In Figure 9, the inner velocity loop is reduced to the transfer function $\frac{V_{s}}{{\hat{V}}_{cs}}$, approximated by a first order dynamic system, with the same bandwidth of the velocity control loop. The force applied to the patient $F_{e}$ is generated by the spring element $k_{s}$, due to the displacement between the motor output ($P_{s}$) and the patient’s hand position ($P_{e}$).

Using the block diagram of Figure 7, the complete equations of the LTIdynamic model for the master (impedance model) and the slave (admittance model) are expressed respectively as:

(4) $$\begin{array}{ccc} Z_{m}V_{h} & = & F_{h}+F_{cm}\\ & = & F_{h}-C_{m}V_{h}-C_{4}e^{-sT_{d}}V_{e} \end{array}$$

(5) $$\begin{array}{ccc} Y_{s}F_{e} & = & -V_{e}+V_{cs}=-V_{e}+{\hat{V}}_{cs}-E_{5}V_{e}\\ & = & -\left(1+E_{5}\right)V_{e}+{E_{1}}^{-1}e^{-sT_{d}}F_{h}-{E_{s}}^{-1}F_{e} \end{array}$$

In Equation (5), if the exchanged force signal from the master to the slave is the master command force $F_{cm}$, precisely $-F_{cm}$, instead of the master contact force $F_{h}$ (as actually happens in the actual implementation; see Figure 5), then the actual LTI dynamic model for the slave can be expressed as:

(6) $$-\left(1+E_{5}\right)V_{e}+{E_{1}}^{-1}e^{-sT_{d}}\left(F_{h}-Z_{m}V_{h}\right)-{E_{s}}^{-1}F_{e}$$

in which Equation (6) differs from Equation (5) only for the term related to $Z_{m}V_{h}$. Therefore, Equation (6) shows that if the impedance of the master device $Z_{m}$ is low and movements are slow (tele-assessment), force $-F_{cm}$ can approximate $F_{h}$, i.e., Equation (6) converges to Equation(5), which explains why $F_{h}$ is the variable sent by the master device to the slave site in Figure 7.

At the slave side, the velocity control loop and the presence of a spring element give the representation of the slave admittance $Y_{s}$ and the slave local position controller $E_{5}$, respectively:

(7) $$\begin{array}{ccc} Y_{s} & = & \frac{s\left(As+1\right)}{k_{s}} \end{array}$$

(8) $$\begin{array}{ccc} E_{5} & = & As \end{array}$$

Notice that $E_{5}$ does not appear as a “control action”, but it is due to the low pass filter of the velocity transfer function. Calculating the values from Equations (4) and (5), the elements of inverse hybrid matrix G are reduced to the following:

(9) $$\begin{array}{cccc} g_{11} & =\frac{1}{Z_{cm}} & g_{12} & =\frac{C_{4}e^{-sT_{d}}}{Z_{cm}}\\ g_{21} & =\frac{{E_{1}}^{-1}e^{-sT_{d}}}{Y_{es}} & g_{22} & =\frac{1+E_{5}}{Y_{es}} \end{array}$$

where $Z_{cm}=Z_{m}+C_{m}$ and $Y_{es}=Y_{s}+{E_{s}}^{-1}$.

### 3.3. Definition of the Control Law

In order to achieve good performance and stability, maximum transparency on each side of the system is considered. Thus, a sub-optimal law of a P-F two-channel teleoperation system, derived from the optimal law for a four-channel impedance-admittance controller, was implemented.

A tele-operation system is ideally transparent if the operator works in real contact with the environment. Considering the MSN diagram block in Figure 6, from an electrical point of view, this means directly connecting the operator to the environment through the two-port block. The interpretation in terms of the MSN matrix (Equation (3)) is given by [39]:

(10) $$G=\left[\begin{array}{cc} Y_{in} & V.\mathrm{Scale}\\ F.\mathrm{Scale} & Z_{out} \end{array}\right]=\left[\begin{array}{cc} 0 & -1\\ 1 & 0 \end{array}\right]$$

If the communication delay $T_{d}$ is negligible, it can be demonstrated that the following control law [40,41]:

(11) $$\left\{\begin{array}{ccc} {E_{1}}^{-1} & = & Y_{es}\\ C_{2} & = & 1+C_{6}\neq 0\\ E_{3} & = & 1+E_{5}\neq 0\\ C_{4} & = & -Z_{cm} \end{array}\right.$$

satisfies Equation (10). However, in the P-F-type two-channel architecture, $E_{3}=C_{2}=0$, so the optimal law for transparency reduces to:

(12) $$\left\{\begin{array}{ccc} {E_{1}}^{-1} & = & Y_{es}=Y_{s}+{E_{s}}^{-1}\\ C_{4} & = & -Z_{cm}=-\left(Z_{m}+C_{m}\right) \end{array}\right.$$

and this does not allow for the elimination of the dynamics from the master and slave, but only an unscaled bilateral transmission of both velocity and force, as investigated further. In addition, due the VS-SEA structure, the channel ${E_{1}}^{-1}$ was imposed equal to ${E_{s}}^{-1}$; also, $Z_{m}$ was not implemented in $C_{4}$. However, being that the acceleration signals are difficult to measure precisely, the channels ${E_{1}}^{-1}$ and $C_{4}$ actually never compensate the master and slave dynamics, but only the local control actions [41], resulting in this final control law:

(13) $$\begin{array}{cccc} R_{\mathrm{SEA}}={E_{1}}^{-1} & =E_{s}^{-1} & C_{4} & =-C_{m} \end{array}$$

At the master side, we assumed a position PD regulator as the master controller, working on a mass-damper system $Z_{m}$, which represents the master device, as expressed in Figure 8, that in turn produces:

(14) $$C_{4}=-C_{m}=-\left(\frac{k_{m}}{s}+b_{m}\right)$$

Without a measure of force $F_{h}$, the master local force controller $C_{6}$ was not implemented.

Moreover, at the other side, the external force loop was regulated by means of a proportional gain Gwith a low-pass filter:

(15) $$R_{\mathrm{SEA}}={E_{1}}^{-1}={E_{s}}^{-1}=\frac{G_{s}}{1+\tau_{s}s}$$

The numeric values of the controller parameters are reported in Table 1.

For the tuning of the system parameters, we followed the strategy of maximizing the bilateral transmitted stiffness, increasing the proportional gain of the master position local controller in a fixed position of the system until instability. Then, we tried to recover stability, either acting on the derivative gain or eventually reducing the proportional gain.

## 4. System Analysis

### 4.1. Transparency

The system transparency can be defined in terms of transmitted impedance at the system’s sides. In fact, the system is perfectly transparent if:

(16) $$\begin{array}{cc} Z_{to} & =\frac{F_{h}}{V_{h}}{|}_{V_{e}^{*}=0}=\frac{F_{e}}{V_{e}}{|}_{V_{e}^{*}=0}=Z_{e} \end{array}$$

(17) $$\begin{array}{cc} Z_{te} & =\frac{F_{e}}{-V_{e}}{|}_{V_{h}^{*}=0}=\frac{F_{h}}{-V_{h}}{|}_{V_{h}^{*}=0}=Z_{h} \end{array}$$

where $Z_{to}$ and $Z_{te}$ are respectively the impedances transmitted to the operator and to the environment in absence of external inputs (see Figure 7) or, considering Figure 6, the impedances seen from the input and from the output without the presence of generators.

For the case presented here, in which the control law (Equation (12)) has been implemented as described in Section 3.3, from Equation (9), the resulting inverse hybrid matrix gives:

(18) $$G=\left[\begin{array}{cc} \frac{1}{Z_{m}+C_{m}} & -\frac{C_{m}e^{-sT_{d}}}{Z_{m}+C_{m}}\\ \frac{{E_{s}}^{-1}e^{-sT_{d}}}{Y_{s}+{E_{s}}^{-1}} & \frac{1+As}{Y_{s}+{E_{s}}^{-1}} \end{array}\right]$$

This equation indicates that the P-F two-channel proposed architecture cannot reach perfect transparency, defined by Equation (10), due to the absence of both channels ch2–ch3 ($C_{2}$, $E_{3}$) and the implementation of the inverse dynamics in the feedforward controllers of channels ch1–ch4 ($E_{1}$, $C_{4}$). Indeed, if the network delay is negligible, the proposed architecture may provide an unscaled version of both velocity and force, especially at low frequencies ($g_{12}\to -1$, $g_{21}\to 1$), but there is no possibility of canceling the dynamics of the master and slave.

### 4.2. Performance

The performance of a tele-operation system can be evaluated in terms of the impedance transmitted to the operator and to the environment. By using Equations (1)–(3), the transmitted impedances of the proposed architecture are:

(19) $$\begin{array}{cc} Z_{to}= & \frac{F_{h}}{V_{h}}{|}_{V_{e}^{*}=0}=\frac{Z_{e}+g_{22}}{g_{11}\left(Z_{e}+g_{22}\right)-g_{12}g_{21}} \end{array}$$

(20) $$\begin{array}{cc} Z_{te}= & \frac{F_{e}}{-V_{e}}{|}_{V_{h}^{*}=0}=g_{22}-\frac{g_{21}g_{12}}{1+g_{11}Z_{h}}Z_{h} \end{array}$$

However, to analyze the performance of the system, the behavior of the system should be studied for an infinite spectrum of impedance, either from the operator side or from the environment side. Then, $Z_{to}$ and $Z_{te}$ were studied for extreme values of $Z_{e}$ and $Z_{h}$ in order to simplify the analysis. This means studying both master and slave performances when they are either in free motion ($Z_{e}=0$ or $Z_{h}=0$) or clamped ($Z_{e}\to \infty$ or $Z_{h}\to \infty$). By using Equation (9), the limits of the perceived-impedance range can be calculated as follows:

(21) $$\begin{array}{ccc} Z_{to,min} & = & Z_{to}{|}_{Z_{e}=0}=\frac{\left(1+As\right)\left(Z_{m}+C_{m}\right)}{\left(1+As\right)+{E_{s}}^{-1}C_{m}e^{-2sT_{d}}}\\ Z_{to,max} & = & Z_{to}{|}_{Z_{e}\to \infty }=Z_{m}+C_{m}\;\\ Z_{te,max} & = & Z_{te}{|}_{Z_{h}\to \infty }=\frac{\left(1+As\right)+{E_{s}}^{-1}C_{m}e^{-2sT_{d}}}{Y_{s}+{E_{s}}^{-1}}\\ Z_{te,min} & = & Z_{te}{|}_{Z_{h}=0}=\frac{1+As}{Y_{s}+{E_{s}}^{-1}} \end{array}$$

Good performance is then characterized by $\left|Z_{t,min}\right|\to 0$ and $\left|Z_{t,max}\right|\to \infty$. In this case, the range of the perceived impedance at the master side considerably depends on the stiffness of the local position controller $C_{m}$. A higher stiffness of this controller allows a better performance at hard contact, with the drawback of degrading performances in free motion and soft contact. Furthermore, the master local controller $C_{m}$ together with the slave local controller ${E_{s}}^{-1}$, due to the implemented control law, influence the sensibility.

At the slave side, the local force feedback ${E_{s}}^{-1}$ directly participates om the sensibility, but altering the maximum perceivable impedance, whilst it improves hard contact along with the master local controller $C_{m}$, through the feedforward control actions. Instead, the local velocity feedback $E_{5}=As$ improves the maximum perceived impedance, but also deteriorates the minimum impedance.

It is worth noticing that in a tele-rehabilitation system for clinical assessments, the movements should be performed at very slow speeds; in this way, referring to stiffnesses rather than impedances appears as a more appropriate approach. From Equation (21), the perceived stiffness can be so obtained:

(22) $$\begin{array}{cc} k_{to,max} & =k_{to}{|}_{k_{e}\to \infty }=\left(b_{m}s+k_{m}\right)+\left(m_{m}s^{2}+c_{m}s\right) \end{array}$$

(23) $$\begin{array}{cc} k_{te,max} & =k_{te}{|}_{k_{h}\to \infty }=\frac{\left(s+As^{2}\right)+\frac{G_{s}}{1+\tau_{s}s}\left(b_{m}s+k_{m}\right)}{\frac{s\left(As+1\right)}{k_{s}}+\frac{G_{s}}{1+\tau_{s}s}} \end{array}$$

Therefore, the maximum perceived stiffnesses in static conditions, that is for s→0, can be expressed as follows:

(24) $$\begin{array}{cccc} k_{to,max}{|}_{s\to 0} & =k_{m} & k_{te,max}{|}_{s\to 0} & =k_{m} \end{array}$$

The maximum perceived stiffness by the clinician and by the patient would be then not infinite, but limited by the proportional gain of the position controller. This result appears as intuitive at the master side, but conversely, it does not look so at the slave side. In fact, its meaning is that in static conditions, the elastic element of the SEA is not perceived at all.

### 4.3. Stability

The stability of an impedance-admittance teleoperation system can be evaluated by Llewellyn’s criterion, which is expressed in terms of the elements of the immittance matrix that describes the MSN system [41,47]:

An LTI two-port network is absolutely stable if and only if:

- $p_{11}$ and $p_{22}$ have no poles in the open right half-plane (RHP)
- any pole of $p_{11}$ and $p_{22}$ on the imaginary axis are simple and have real positive residuals
- $\eta_{p}\left(\omega \right)=-\cos\left(∠p_{12}p_{21}\right)+2\frac{Re\left(p_{11}\right)Re\left(p_{22}\right)}{\left|p_{12}p_{21}\right|}\geq 1$ and $Re\left(p_{11}\right)\geq 0$

The absolute stability only depends on the network parameters, independently from the operator’s and environment’s linearity. A system that is not absolutely stable is called potentially unstable, that is there exists a particular passive pair of operator and environment that may destabilize the system. Obviously, such a system is not necessarily unstable.

For the P-F proposed teleoperation system, using the absolute stability condition with the inverse hybrid matrix G, the stability network parameter $\eta_{pf}$ can be evaluated, getting the following expression:

(25) $$\begin{array}{cc} \eta_{pf}\left(\omega \right)=\eta_{pf1} & +\eta_{pf2}=\\ =-\cos\left(∠-\frac{{E_{s}}^{-1}C_{m}e^{-2j\omega T_{d}}}{Y_{es}Z_{cm}}\right) & +2\frac{Re\left(\frac{1}{Z_{cm}}\right)Re\left(\frac{1+As}{Y_{es}}\right)}{\left|-\frac{{E_{s}}^{-1}C_{m}e^{-2j\omega T_{d}}}{Y_{es}Z_{cm}}\right|} \end{array}$$

If ideally the inverse dynamics at each side of the system had been integrated into the feedforward controllers ($C_{4}$, ${E_{1}}^{-1}$) and, furthermore, the time delay had been negligible, in order to achieve the absolute stability, it would have been enough that $Re\left(\frac{1}{Z_{cm}}\right)Re\left(\frac{1+As}{Y_{es}}\right)\geq 1$ for any frequency. However, in our case, the analysis is more complex, and the absolute stability may be guaranteed in only a certain range of frequencies and in the presence of small delays. Indeed, it can be proven that the second term $\eta_{pf2}\to 0$ at low and high frequencies, when the control parameters and system models are defined as in the case considered here. On the other hand, the larger the delay, the more difficult is the design of the controllers, as the first term of Equation (25) rapidly changes its sign with the frequency *ω*.

**Figure 10:**
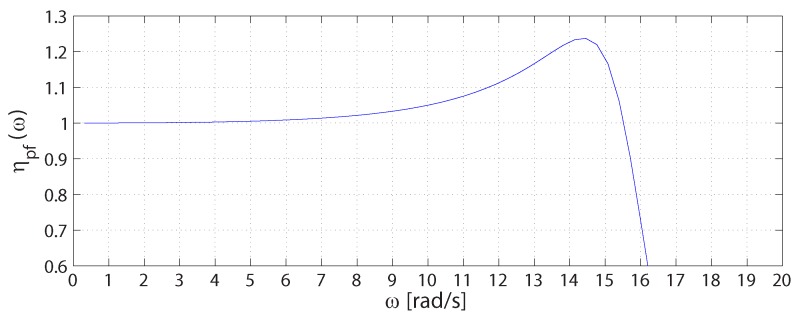
Curve of absolute stability with the parameter values defined in Subsection III-C, but with the proportional gain $k_{m}$ of the master position controller set to 20% of the actual one.

Experimentally, we found that for the considered system, stable control systems can be designed up to a delay of 30 ms. However, the evaluation of Equation (25) shows that with the parameter values defined in Section 3.3, the system is always absolutely stable for overall communication delays up to 30 ms in a range of frequency of about 0÷2.5 Hz only, as shown in Figure 10, but with a value for the proportional gain of the master position controller ($k_{m}$) assumed to be up to 20% of the actual one. This result is not misleading since a system that is not absolutely stable is not necessarily unstable. Anyhow, this difference may be due to the model assumed here for the slave device, which actually does not account for the friction, whereas it is known that friction causes steady effects on such a system. Indeed, this could explain an allowable more increased value for $k_{m}$ in the actual system implementation.

Otherwise, it would be always possible to extend the region of absolute system if for example $k_{m}$ were reduced, with the drawback of the maximum transmissible stiffness consistently cut down. However, in our context (mostly remote assessment), the transmissible stiffness at very low frequencies is the most relevant requirement when stability is guaranteed; for this reason, we opted for a tighter master regulator.

## 5. Preliminary Experimental Tests

### 5.1. Protocol

In order to preliminarily verify the experimental behavior of the developed device, a therapist replicated a practical session of the remote assessment of the state of the hand. Specifically, she/he applied to three healthy male subjects four of the total clinical tests defined in Section 2.1, i.e., the test of the range of motion (passive and active) and the muscular/resistance force tests. The clinician instructed subjects to move the hand slowly as in an actual assessment session, which we measured about 20÷30 ${}^{\circ }$/s. The tests of range of motion were repeated three times consecutively (i.e., three consecutive closing/opening movements of the hand), and the motion of the subject hand was limited by using two elements, which blocked hands to simulate a reduced range of motion. For the muscular and resistance tests, both flexion and extension movement were repeated two times after some seconds, during which a subject moved the hand in order to execute the next repetition at a random hand position. In addition to such tests, a safety test was performed by the clinician for each subject, by releasing suddenly the master handle during one repetition of either flexion or extension resistance test.

Healthy subjects were used instead of patients, because a first evaluation of the system performance could be obtained only with healthy hands and in the absence of spasticity. Before the experiments, the system was conveniently tuned.

### 5.2. Results

In the following, we present the results of the preliminary experimental tests. Table 2 summarizes the results by showing an index of performance both for each subject and for each test repetition. For the first type of tests (ROM), this index consists of a measure of the angular range of motion, considered at the MCP joint, for both clinician and subjects; for the muscular and resistance tests, the index represents the measure of the hand stiffness.

In addition to such tests, two technical tests were also performed to measure the maximum perceived stiffnesses, which were compared to the theoretical values found in the system analysis.

#### 5.2.1. Active and Passive Range-Of-Motion Tests

During a conventional passive ROM test, the clinician identifies movements or positions that cause pain or discomfort to the patient. Similarly, during active ROM evaluations, the patient is asked to move his or her own fingers as much as possible.

A remote passive ROM test could be conceived of as a traditional evaluation in which the therapist moves the prosthetic hand at the master side while the patient relaxes the hand plugged into the slave orthosis. Then, the clinician may determine the range of motion of the hand fingers by either experiencing an increased force at the handle or by using additional feedbacks as an audio-video streaming of the patient assessment could provide. Similarly, a remote active ROM test may be performed.

**Figure 11:**
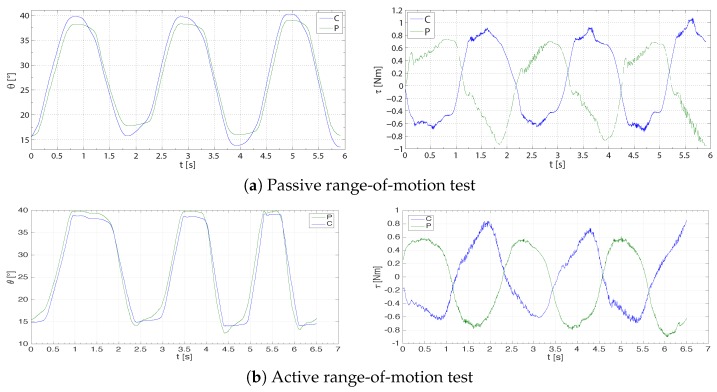
Example of position (**left**) and torque (**right**) tracking performance for the position-force (P-F) two-channel controller operating in contact with a healthy subject during a trial of a passive range-of motion test (**top**) and an active range-of motion test (**bottom**), respectively. *τ* (Nm) is the torque, positive for the clinician (C, blue) extension and for the subject (P, green) flexion, respectively. *θ* (${}^{\circ }$) is the angle, which increases with flexion.

The fundamental requirement for this kind of tests, as stated also in Section 2.1, is a good bilateral tracking performance of the system. Figure 11 shows an example of tracking performance of the proposed system with an input movement provided by either the clinician at the master side (passive ROM test, Figure 11a) or a subject at the slave side (active ROM test, Figure 11b). In the first case, a low force resistance is mostly due to the relaxed hand of the healthy subject. In the latter, the resistance is due to the clinician that grasps the prosthetic hand following the subject movement. The force resistance is maximum at the limits of the movement, in which the clinician moves over the limit of the subject hand (passive ROM) or keeps fixed the master handle when the subject changes the movement direction, i.e., from flexion to extension or vice versa (active ROM). This is also evidenced by different values of the range of motion by comparing one side of the teleoperation system to the other, as shown in Table 2a.

#### 5.2.2. Muscular and Resistance Tests

**Figure 12:**
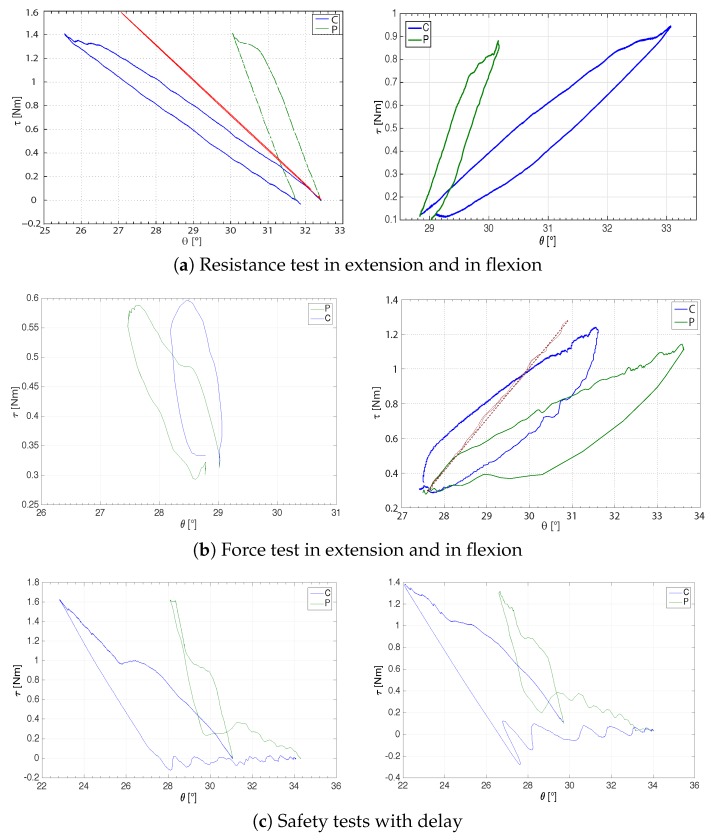
Examples of the torque-angle diagram during resistance (**top**) and muscular tests (**middle**) either in extension (**left**) or flexion (**right**). In addition, two examples of safety tests in the presence of a communication delay are shown during resistance tests in extension (**bottom**). The curves for both clinician (C, blue) and subject (P, green) are reported in the positive half-right plane. *τ* (Nm) indicates the torque; *θ* (${}^{\circ }$) is the angle, which increases with flexion. The maximum-perceived-stiffness curves (see Section 5.2.3) are shown in red.

These experimental tests consist of flexion-extension movements to evaluate the state of stiffness of the hand. In Figure 12, the results of some flexion-extension tests are depicted as torque-angle diagrams.

In the first exercise, shown in Figure 12a for one subject, the clinician tried to move the subject’s hand, namely open (left figure) or closed (right figure). Consider firstly the extension case (left figure). The clinician extended up, while the subject was asked to stay still with his hand at the current position. This exercise simulates a possible remote evaluation of the muscular resistance or the flaccidity/stiffness of the patient’s hand. Then, while the clinician was trying to extend (upper curve) and release (lower curve) the subject’s hand, a fair reproduction of the force could be needed, as that which seems to be obtained by the proposed master-slave system. The pushing and releasing curves differed from each other (hysteresis) because the subject was not able to remain with his hand perfectly fixed, yet he moved it while the clinician was performing the forced extension (slightly changing his hand stiffness), leading to a releasing curve with lower force values at the same angle values. In fact, referring to the scheme of Figure 7, the generated force by the master and slave (approximately equal to the one exchanged by the operator himself, especially if the impedances are neglected) is proportional to the difference between the slave and the master positions. Because of this, a lower horizontal offset between the subject and the clinician curves resulted in a consistent reduction of the perceived force. In the opposite movement (flexion test), i.e., the clinician forces the subject’s hand in order to flex it; similar results can be seen in Figure 12a (right): the stiffness was different at the two sides; precisely, one was a scaled version of the other. This was mainly due to the fact that the operators were performing different tasks, and the maximum reachable stiffness was limited. Indeed, the subject stiffened his hand (higher stiffness) in order to hold it fixed as much as possible. The therapist softened her/his muscles (lower stiffness) and tried to create a wider offset between the position input and the actual master position. Similarly, the therapist can apply a higher force to generate the same transmitted force either to the master or to the slave.

Figure 12b shows instead two active movements performed by a subject, who was asked to extend (left figure) or flex (right figure) the fingers of his hand, trying to overcome the therapist resistance, who aimed to maintain his position. The results are again comparable with the previous ones by switching the subject, who performed the “active” task in this case, with the clinician.

Different values of stiffness at one system side if compared to the other side are also visible in Table 2b. Specifically, the table shows that the stiffness assumes lower values at the system side where the active task was performed (flexion or extension), with respect to those at the side of the passive task (keeping the hand in the same position). These results highlight that these flexion-extension tests will have to be repeated with patients in order to actually understand if the remote sensations of the therapist regarding the state of patient’s hand are comparable to those of a close interaction during an actual practical session. The results of such tests will be crucial to explain how to tune the control system in order to achieve a clear and valid tele-assessment system.

Table 2b allows us to make also a few other observations. The first one is that stiffness appears largely variable in most cases by comparing the first with the second repetition for either the clinician or the subject, regardless of the type of test (resistance or muscular). This is mainly due to a different angular position of the phalanges of the hand fingers around which either the flexion or extension test was performed. In fact, the force of the fingers is related to the configuration of the phalanges [48,49]. In this way, flexion and extension tests will have to be replicated with patients and for different angular positions in order to test the performance of the system tuning in different configurations. A last mention should be made for the comparison between resistance and muscular tests. Due to the design of experiment that expected test repetitions at random positions, i.e., not the same angular positions for the muscular tests with respect to those of resistance tests, as well as due to a limited perceived stiffness, we cannot compare the results of these two tests. It can be said that healthy subjects should present similar force values in the two tests, neglecting individual differences [48,49], but the variation of the angular position values of the phalanges in the two cases may cause small differences in stiffness. However, the comparison would assume more importance if the subjects were post-stroke patients. In fact, in that case, we could expect differences in force, as well position that could result in larger differences in stiffness by comparing the two cases around the same angular positions, due to impairments following stroke. Moreover, the force would be more reduced [50], as well as the values of stiffness, which make this system more suitable to find differences.

#### 5.2.3. Safety Tests and Maximum Perceived Stiffness

The next tests regarded safety. During an extension test, the clinician suddenly released the master while a subject was applying a force, with a random time delay of 20÷30 ms through the communication line. Figure 12c show two examples of the resulting curves, obtained with two different subjects. Notice how a small amplitude vibration was triggered in the system and immediately adsorbed.

Stability and performance showed equivalent results either in the presence or without a small time delay, confirming the effectiveness of the tuning control parameters.

In order to evaluate the performance of the teleoperation system, some tests were also conducted by recording the maximum transmitted impedances. These tests were performed under static conditions. Therefore, we simulated an infinite-impedance condition by moving either the prosthetic hand or the orthosis’ end-effector to a certain position and by turning off the opposite device. The resulting torque-angle curves are shown in Figure 12 (red curves), more precisely in Figure 12a,b, respectively.

Particularly interesting has been the estimation of the maximum stiffnesses transmittable to the clinician ($k_{to,max}$) and to the possible patient ($k_{te,max}$). Therefore, referring to Figure 12a, the maximum stiffness seen from the operator can be computed from the line slope of the master torque-angle curve and resulted as $k_{to,max}=\frac{\Delta \tau }{\Delta \theta }≃17$ Nm/rad. Furthermore, the maximum stiffness seen from the environment resulted as $k_{te,max}=\frac{\Delta \tau }{\Delta \theta }≃17$ Nm/rad.

These values confirm the theoretical results on the system performance explained in Section 4.2, since the maximum stiffnesses at both sides approximately assumed the same value of the proportional gain of the master position regulator ($k_{m}$), as reported in Table 1.

## 6. Conclusions

In this paper, a proof of concept has been presented of a new application for real-time bilateral haptic interfaces, which may extend the use of real-time teleoperation systems to the remote motor and functional clinical assessment of the hand, in patients with neurological impairments. We believe that this kind of application could have an important role, since the current trend is to bring the rehabilitation treatment to the patient’s home.

The paper has reported an example of a bilateral tele-assessment architecture, obtained by adapting a pre-existent stand-alone hand rehabilitation system, thus avoiding a new ad hoc device. Technical specifications, methods and procedures have been suggested, outlining a design framework for this kind of application.

A two-channel bilateral control system architecture has been designed and implemented. The system was preliminarily tested in a replication of a bilateral haptic interaction for the remote assessment of the state of the hand of healthy subjects, limited to the range of motion and flexion/extension tests. In these experiments, the system has proven to be a reliable framework. Furthermore, it has shown the capability of maintaining the overall stability, even in the presence of small network delays.

The theoretical analysis has shown that the level of transparency and performance was limited by the use of a two-channel architecture. Additionally, the absence of a local force controller in the master device further limited the achievable performance and stability ranges. This suggests that future versions of such a system should rely on a four-channel architecture.

Finally, further studies should investigate the extended case of master/slave devices with more degrees of freedom.

## Figures and Tables

**Table 1:** Control parameters of the master and the slave devices.

Master Parameters			Slave Parameters		
Par.	Units	Values	Par.	Units	Value
$k_{m}$	Nm/rad	17.38	$k_{s}$	Nm/rad	22.93
$b_{m}$	Nm/(rad/s)	0.0178	*A*	s	0.2000
$m_{m}$	kgm${}^{2}$	0.000588	*τ*	s	0.0083
$c_{m}$	Nm/(rad/s)	0.009	*G*	(rad/s)/Nm	2.18

**Table:** (**a**) Range-of-motion (ROM) tests.

Subj.	Passive ROM Test						Active ROM Test					
	Clinician ROM [${}^{\circ }$]			Subject ROM [${}^{\circ }$]			Clinician ROM [${}^{\circ }$]			Subject ROM [${}^{\circ }$]		
	$\Delta \theta_{o,1}^{p}$	$\Delta \theta_{o,2}^{p}$	$\Delta \theta_{o,3}^{p}$	$\Delta \theta_{e,1}^{p}$	$\Delta \theta_{e,2}^{p}$	$\Delta \theta_{e,3}^{p}$	$\Delta \theta_{o,1}^{a}$	$\Delta \theta_{o,2}^{a}$	$\Delta \theta_{o,3}^{a}$	$\Delta \theta_{e,1}^{a}$	$\Delta \theta_{e,2}^{a}$	$\Delta \theta_{e,3}^{a}$
S1	24.08	25.87	26.28	22.42	22.24	22.90	22.43	22.25	24.50	25.13	26.35	29.27
S2	22.31	23.58	23.15	19.34	19.37	20.94	21.57	22.58	21.83	26.68	26.85	24.89
S3	26.02	25.83	24.46	22.11	22.31	21.68	22.21	23.08	23.41	25.76	26.67	26.31

**Table:** (**b**) Resistance and muscular tests.

Subj.	Resistance Test								Muscular Test							
	Extension Stiffness [Nm/rad]				Flexion Stiffness [Nm/rad]				Extension Stiffness [Nm/rad]				Flexion Stiffness [Nm/rad]			
	Clinician		Subject		Clinician		Subject		Clinician		Subject		Clinician		Subject	
	$k_{o,1}^{re}$	$k_{o,2}^{re}$	$k_{e,1}^{re}$	$k_{e,2}^{re}$	$k_{o,1}^{rf}$	$k_{o,2}^{rf}$	$k_{e,1}^{rf}$	$k_{e,2}^{rf}$	$k_{o,1}^{me}$	$k_{o,2}^{me}$	$k_{e,1}^{me}$	$k_{e,2}^{me}$	$k_{o,1}^{mf}$	$k_{o,2}^{mf}$	$k_{e,1}^{mf}$	$k_{e,2}^{mf}$
S1	13.46	10.73	46.72	35.44	11.60	11.87	37.11	31.70	35.01	23.89	12.80	14.21	12.94	13.88	8.10	7.53
S2	6.51	8.12	10.43	12.61	8.43	10.66	16.78	13.53	18.86	21.27	11.14	13.33	12.28	18.69	6.5	9.38
S3	8.25	8.42	13.05	13.12	12.65	10.98	27.49	22.30	22.17	30.81	10.05	15.03	20.43	12.95	9.8	8.25
